# Widespread transcriptional disruption of the microRNA biogenesis machinery in brain and peripheral tissues of individuals with schizophrenia

**DOI:** 10.1038/s41398-020-01052-5

**Published:** 2020-11-04

**Authors:** Romain Rey, Marie-Françoise Suaud-Chagny, Jean-Michel Dorey, Jean-Raymond Teyssier, Thierry d’Amato

**Affiliations:** 1 INSERM, U1028; CNRS, UMR5292, Lyon Neuroscience Research Center, Psychiatric Disorders: from Resistance to Response Team, 69000 Lyon, France; 2 University Lyon 1, 69000 Villeurbanne, France; 3 Schizophrenia Expert Centre, Le Vinatier Hospital, Bron, France; 4 Department of Old Age Psychiatry, Le Vinatier Hospital, Bron, France; 5 Department of Genetics and Laboratory of Molecular Genetics, University Hospital, Dijon, France

**Keywords:** Epigenetics in the nervous system, Biomarkers

## Abstract

In schizophrenia, altered transcription in brain and peripheral tissues may be due to altered expression of the microRNA biogenesis machinery genes. In this study, we explore the expression of these genes both at the cerebral and peripheral levels. We used *shiny*GEO application to analyze gene expression from ten Gene Expression Omnibus datasets, in order to perform differential expression analyses for eight genes encoding the microRNA biogenesis machinery. First, we compared expression of the candidate genes between control subjects and individuals with schizophrenia in postmortem cerebral samples from seven different brain regions. Then, we compared the expression of the candidate genes between control subjects and individuals with schizophrenia in three peripheral tissues. In brain and peripheral tissues of individuals with schizophrenia, we report distinct altered expression patterns of the microRNA biogenesis machinery genes. In the dorsolateral prefrontal cortex, associative striatum and cerebellum of individuals with schizophrenia, we observed an overexpression pattern of some candidate genes suggesting a heightened miRNA production in these brain regions. Additionally, mixed transcriptional abnormalities were identified in the hippocampus. Moreover, in the blood and olfactory epithelium of individuals with schizophrenia, we observed distinct aberrant transcription patterns of the candidate genes. Remarkably, in individuals with schizophrenia, we report DICER1 overexpression in the dorsolateral prefrontal cortex, hippocampus and cerebellum as well as a congruent DICER1 upregulation in the blood compartment suggesting that it may represent a peripheral marker. Transcriptional disruption of the miRNA biogenesis machinery may contribute to schizophrenia pathogenesis both in brain and peripheral tissues.

## Introduction

Schizophrenia (SZ) affects approximately 1% of the worldwide population and is responsible for a tremendous burden on society^1^. Subjects with SZ have a life expectancy of approximately 20 years below that of the general population^2^. Indeed, SZ is responsible for a dramatic increase in mortality due to suicide but also to somatic diseases (especially metabolic, cardiovascular pathology, and cancers)^2^. In this respect, SZ is now considered as a systemic disease in which pathological processes take place not only at the cerebral level but also in peripheral tissues^3,4^. Despite its high prevalence and major clinical impact, SZ pathogenesis remains elusive. However, there is strong evidence that the disorder is caused by the interplay of environmental and genetic factors^5^.

New insights in schizophrenia pathogenesis have been brought by the advent of microarray technologies and more recently by transcriptome sequencing^6–8^. High-throughput gene expression studies have constantly reported the aberrant transcription of numerous genes in brain and peripheral tissues of individuals with SZ (SZ individuals). To date, most findings from human postmortem brain tissues, peripheral tissues as well as animal models have characterized differential expression of genes involved in presynaptic function, neurotransmission, signaling, myelination, neural development and migration, immune/inflammatory mechanisms, energy production, and response to oxidative stress^6–9^. Altogether, these studies indicate that SZ is associated with a global disturbance across many genes and that SZ-associated gene expression patterns correspond with functional pathways. This suggests that a complex dysregulation of gene expression may be involved in SZ pathogenesis.

To date, the majority of studies investigating the underlying mechanisms driving these transcriptional abnormalities have focused on alterations in transcription factors^10^, gene promoter elements^11^, DNA methylation^12^, or post-translational histone modifications^13^. However, there is now increasing evidence indicating that post-transcriptional influences on gene expression mediated by noncoding RNAs are implicated in SZ pathogenesis^14–18^. Among the different classes of noncoding RNAs, microRNAs (miRNAs) are the most widely studied. In the central nervous system, miRNAs are involved in the regulation of many essential mechanisms such as neuronal differentiation, adult neurogenesis, or synaptic plasticity^19–21^. While some miRNAs can regulate the expression of one specific target, others can be considered as master regulators of a process since they have the ability to control the expression levels of hundreds of genes at the same time^22^. Moreover, many types of miRNAs control their targets cooperatively^23^. Indeed, since as much as 60% of the human protein-coding genes exhibit at least one conserved miRNA-binding site, it has been suggested that miRNAs may regulate the majority of them^24^. Functionally, miRNAs regulate gene expression through the binding to target sites in the 3′-untranslated region of mRNAs; in this way they can either prevent mRNA translation into protein due to steric hindrance of the protein synthesis machinery or target the mRNA for enzymatic degradation^23^.

**Fig. 1:**
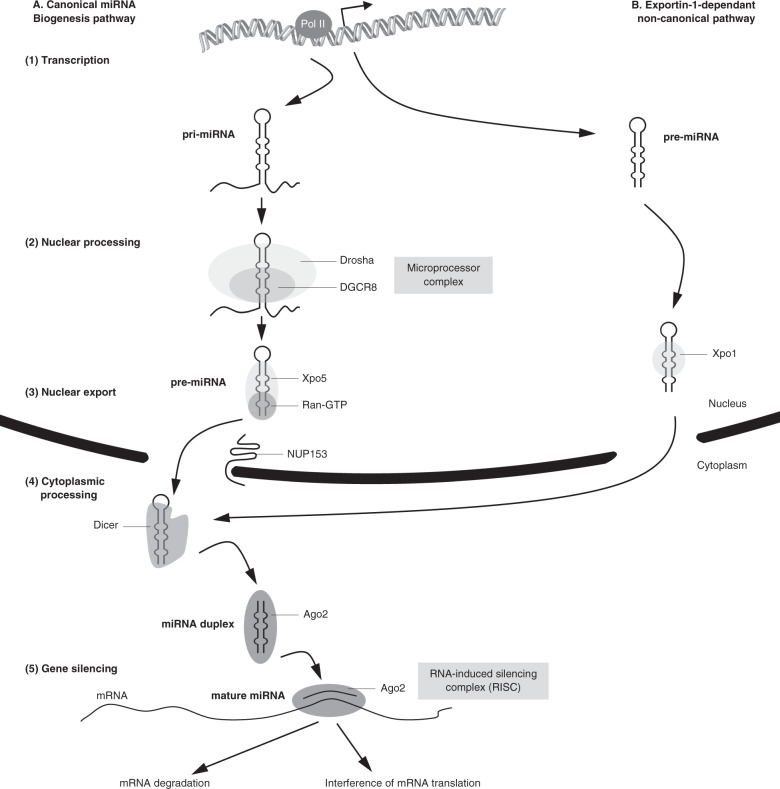
Canonical and noncanonical pathways of miRNA biogenesis. **A** Canonical miRNA biogenesis pathway: (1) Transcription: a primary miRNA (pri-miRNA) is transcribed by the RNA polymerase II. (2) Nuclear processing: the Microprocessor complex, composed of the RNase III Drosha and its cofactor DGCR8, initiates the maturation process and releases a precursor miRNA (pre-miRNA). (3) Nuclear export: the pre-miRNA is recognized and exported to the cytoplasm by the Exportin-5 (Xpo5)/Ran-GTP transporter interacting with NUP153. (4) Cytoplasm processing: the pre-miRNA undergoes a second processing by the RNase III Dicer. The generated miRNA duplex is then loaded into an Argonaute protein (Ago2) which preferentially ejects one strand and retains the mature miRNA. (5) Gene silencing: Ago2 and the mature miRNA form the RNA-induced silencing complex (RISC). RISC recognizes target mRNA by paring the 5′-end of the miRNA molecule with a partially complementary sequence in the 3′-untranslated region of target mRNAs. **B** Exportin-1-dependant noncanonical pathway: a pre-miRNA is generated directly through transcription, exported by exportin-1 (Xpo1) and undergoes the usual cytoplasm processing.

Most of miRNAs are produced by the canonical miRNA biogenesis pathway. This molecular pathway relies on a miRNA biogenesis machinery (BM) constituted of several genes encoding enzymes and cofactors implicated in the transcription, nuclear processing, export and maturation of miRNAs (Fig. 1). Notably, various polymorphisms located in the miRNA BM genes have been associated with SZ risk^25–28^, and microarray studies in brain and peripheral tissues of SZ individuals have reported altered expression of several miRNAs suggesting a broader disruption of the miRNA BM^14,18,29^. Previously, Beveridge et al. proposed the hypothesis that schizophrenic disorders are associated with a dysregulation of the miRNA BM in the cerebral cortex^30^. To date, only four human studies have quantified the expression of genes coding for the miRNA BM in postmortem brain samples^30,31^ and peripheral blood^11,32^ of SZ individuals. To our knowledge, no study has systematically explored the expression of the main miRNA BM genes in various brain regions or peripheral tissues of SZ individuals.

In this study, we used the bioinformatic application *shiny*GEO to analyze gene expression from eight Gene Expression Omnibus (GEO) datasets, in order to perform differential expression analyses for eight genes encoding the canonical miRNA BM. Firstly, we compared expression of the candidate genes between healthy controls (HC) and SZ individuals in postmortem cerebral samples from seven different brain regions. Secondly, we compared the expression of the candidate genes between HC and SZ individuals in three peripheral tissues. Thirdly, we tried to replicate our findings by analyzing the expression levels of the identified differentially expressed genes in independent validation datasets. Finally, to evaluate the neurobiological relevance of the changes identified in peripheral tissues, we compared them to those observed in brain regions.

## Material and methods

### Search and inclusion criteria of primary datasets

The GEO database is a public repository, which archives and freely distributes microarray functional genomic data from control subjects and patients suffering from various disorders, along with demographic, clinical and quality data^33^. GEO datasets thus constitute a valuable resource for identifying biomarkers of diseases.

With the aim to identify altered expression of the miRNA BM genes both at the central and peripheral level in SZ individuals, we explored GEO database for datasets providing microarray expression results from SZ individuals and HC. Microarray datasets related to SZ, were searched in NCBI GEO database using the following search terms: “schizophrenia”[All Fields] AND “Homo sapiens”[porgn] AND “gse”[Filter] AND “Expression profiling by array”[Filter]. We only included datasets from original studies (i) involving brain or peripheral tissues samples from SZ individuals and HC and (ii) using the Affymetrix Human Genome U133 plus 2.0 (HG-U133_Plus_2) or Affymetrix Human Gene 1.0 ST (Human Gene 1.0 ST) or Affymetrix Human Gene 1.1 ST (Human Gene 1.1 ST) arrays. Each of these three chips can technically interrogate the expression of all the candidate genes included in the present study. Moreover, Human Gene 1.0 ST and Human Gene 1.1 ST arrays exhibit comparable detection thresholds and are highly concordant with HG-U133_Plus_2 array^34,35^.

### Included primary datasets

In this study, eight independent GEO datasets were included^36–43^, providing data from seven different brain regions relevant to SZ^44–47^ (dorsolateral prefrontal cortex (DLPFC) (BA46), anterior prefrontal cortex (BA10), parietal cortex, superior temporal cortex (BA22), hippocampus, associative striatum, and cerebellum) and three peripheral tissues (peripheral blood mononuclear cells (PBMCs), olfactory epithelium, and skin fibroblasts). The original studies from which the primary datasets were obtained are presented in Supplementary Table 1. It should be noticed that postmortem brain and peripheral tissues samples were collected from different subjects.

### Search and inclusion criteria of validation datasets

With the aim to replicate our main findings, validation datasets were searched in GEO database with identical search terms as those used in the first step of the study. For validation datasets, we selected GEO datasets from original studies (i) involving SZ individuals and HC, (ii) with samples from a brain region or a peripheral tissue in which altered transcription was observed in the primary datasets (i.e., DLPFC (BA46), associative striatum, hippocampus, cerebellum, blood compartment, and olfactory epithelium), (iii) using chips able to technically interrogate the expression of the differentially expressed genes identified in the primary datasets. Additionally, included validation datasets had to meet the following criteria: (i) a primary dataset and its validation counterpart must be derived from independent samples and (ii) the sample size of the validation dataset must be equal to or larger than that of its primary counterpart.

### Included validation datasets

At the cerebral level, we included one validation dataset^48^ providing expression data from the DLPFC (BA46) of SZ individuals and HC. Regarding the blood compartment, one validation dataset^49^ was included, providing expression data from the whole blood of SZ individuals and HC. The original studies from which the validation datasets were obtained are presented in Supplementary Table 2.

### Demographic characteristics and quality data

For each primary and validation GEO dataset, the quality data of the samples and the demographic characteristics of the subjects, who provided brain or peripheral tissue are summarized in Tables 1 and 2, respectively. For each GEO dataset providing data from postmortem brain samples, there was no significant difference in age, sex distribution, postmortem interval (PMI), and RNA integrity number (RIN) between the SZ individual and HC groups. For the brain pH, a significant difference was only observed in the anterior prefrontal cortex BA10 (*p* = 0.019) and in the superior temporal cortex (*p* = 0.002). For each GEO dataset providing data from peripheral tissues, there was no significant difference in age distribution between the SZ patient and HC groups. For the sex distribution, a significant difference was only observed in the whole blood (*p* = 0.0001).

**Table 1:** Demographic and quality characteristics of the postmortem brain samples.

Brain region	Subjects (n)	Age (mean ± SD)			Gender (M/F)			Post-mortem interval (mean ± SD)			pH (mean ± SD)			RIN (mean ± SD)		
		Control	Schizophrenia	*p*-value^a^	Control	Schizophrenia	*p*-value^a^	Control	Schizophrenia	*p*-value^a^	Control	Schizophrenia	*p*-value^a^	Control	Schizophrenia	*p*-value^a^
**Primary datasets**																
DLPFC (BA46)	19 HC/15 SZ	48.1 ± 10.6	46 ± 8.6	0.549	10/9	7/8	1	19.5 ± 5.1	18.9 ± 6.7	0.779	6.6 ± 0.2	6.5 ± 0.4	0.551	7.8 ± 0.6	7.6 ± 0.7	0.06
Hippocampus	18 HC/15 SZ	48.2 ± 10.9	45.7 ± 8.8	0.493	9/9	9/6	0.728	19.4 ± 5.2	19.4 ± 7.2	0.775	6.6 ± 0.2	6.4 ± 0.3	0.358	7.4 ± 0.6	6.5 ± 0.5	0.51
Associative striatum	18 HC/18 SZ	48.4 ± 10.8	45 ± 8.8	0.301	10/8	10/8	1	19.8 ± 5.1	19.9 ± 7.1	0.797	6.6 ± 0.2	6.5 ± 0.4	0.337	8.2 ± 0.7	7.9 ± 0.8	0.454
Anterior PFC (BA10)	23 HC/28 SZ	69 ± 21.6	73.3 ± 15.2	0.411	13/10	19/9	0.561	9.9 ± 4.4	8.7 ± 7.0	0.609	6.5 ± 0.3	6.1 ± 0.2	**0.019**	-	-	
Cerebellum	50 HC/44 SZ	45.8 ± 9.3	43.2 ± 9.5	0.182	31/19	32/12	0.283	-	-		6.5 ± 0.3	6.4 ± 0.2	0.776	-	-	
Parietal cortex	50 HC/51 SZ	45.5 ± 9.0	42.6 ± 9.9	0.132	35/15	37/14	0.828	-	-		6.5 ± 0.3	6.4 ± 0.3	0.631	-	-	
Superior temporal cortex (BA22)	19 HC/23 SZ	67.7 ± 22.2	72.2 ± 16.9	0.462	10/9	13/10	1	9.1 ± 4.3	7.1 ± 5.7	0.516	6.5 ± 0.3	6.2 ± 0.2	**0.002**	-	-	
**Validation dataset**																
DLPFC (BA46)	29 HC/30 SZ	44.7 ± 16.1	43.4 ± 16.9	0.759	24/5	24/6	1	40.5 ± 14	39.1 ± 11.9	0.695	6.3 ± 0.2	6.2 ± 0.2	0.199	-	-	

*DLPFC* dorsolateral prefrontal cortex, *PFC* prefrontal cortex, *BA* Brodmann area, *RIN* RNA integrity number, *HC* healthy controls, *SZ* individuals with schizophrenia.

^a^Unpaired t-tests and Fisher exact tests were conducted to assess group differences for continuous and discrete variables, respectively.

**Table 2:** Demographic characteristics of the peripheral tissues samples.

Peripheral tissue	Subjects (*n*)	Age (mean ± SD)			Gender (M/F)		
		Control	Schizophrenia	*p*-value^a^	Control	Schizophrenia	*p*-value^a^
**Primary datasets**							
Blood (PBMCs)	29 HC/43 SZ	23.02 ± 4.03	23.90 ± 4.08	0.378	29/0	43/0	1
Olfactory epithelium	19 HC/19 SZ	39.7 ± 11.6	38.9 ± 11.4	0.829	13/6	13/6	1
Skin fibroblasts	20 HC/20 SZ	48.4 ± 12.2	44.6 ± 12.7	0.340	9/11	10/10	0.758
**Validation dataset**							
Blood (whole blood)	96 HC/106 SZ	39.3 ± 14.2	39.6 ± 10.7	0.877	42/54	76/30	1 × 10^−4^

*PBMCs* peripheral blood mononuclear cells, *HC* healthy controls, *SZ* individuals with schizophrenia

^a^Unpaired *t*-tests and Fisher exact tests were conducted to assess group differences for continuous and discrete variables, respectively.

### Candidate genes

The selected candidate genes code for the main components of the canonical miRNA BM: DROSHA, DGCR8, RAN, XPO5, NUP153, DICER1, and AGO2^50^. Additionally, we included XPO1 which is involved in an alternative, noncanonical pathway^51^ (Fig. 1). Expression of RAN, XPO5, NUP153, and XPO1 has never been systematically explored in previous studies on psychiatric disorders although these genes are involved in miRNAs export to the cytoplasm, which constitutes an essential step in the miRNA maturation process.

### Ethical statement

All the data used in this project were acquired in previous studies, all of which conformed to ethical standards^36–43,48,49^.

### Statistical analysis

XLSTAT software (XLSTAT 2017: Data Analysis and Statistical Solution for Microsoft Excel. Addinsoft, Paris, France, 2017) was used to perform statistical analyses. For each included GEO dataset, demographic characteristics of subjects and quality data of samples in HC and SZ patient groups were compared using unpaired *t*-test (for age, postmortem interval, brain pH, and RNA integrity number), and Fisher exact test (for gender).

For each tissue, we used *shiny*GEO application to realize differential expression analysis between SZ individuals and HC^52^. *shiny*GEO is implemented using R [https://www.r-project.org/] and shiny [http://shiny.rstudio.com/]. *shiny*GEO downloads processed gene expression datasets from GEO using GEOquery package^53^, gene expression values are log2-transformed to stabilize the variance. Descriptive Statistics of log2(candidate gene expression) values in brain and peripheral tissues are provided in Supplementary Table 3. Normal distribution was tested using Shapiro–Wilk test. For differential expression, exact *p*-values were calculated using unpaired, two-tailed *t*-tests. When a deviation from normality was detected, exact *p*-values were calculated using unpaired, two-tailed Mann–Whitney *U*-tests. In case of violation of the equal variance assumption, exact *p*-values were calculated using unpaired, two-tailed Welch tests. The resulting *p*-values have been adjusted by the Benjamini and Hochberg’s approach to control the false discovery rate. Gene expression comparisons were considered to be statistically significant for adjusted *p*-values < 0.05.

## Results

### Differential expression analysis in the brain tissues

**Fig. 2:**
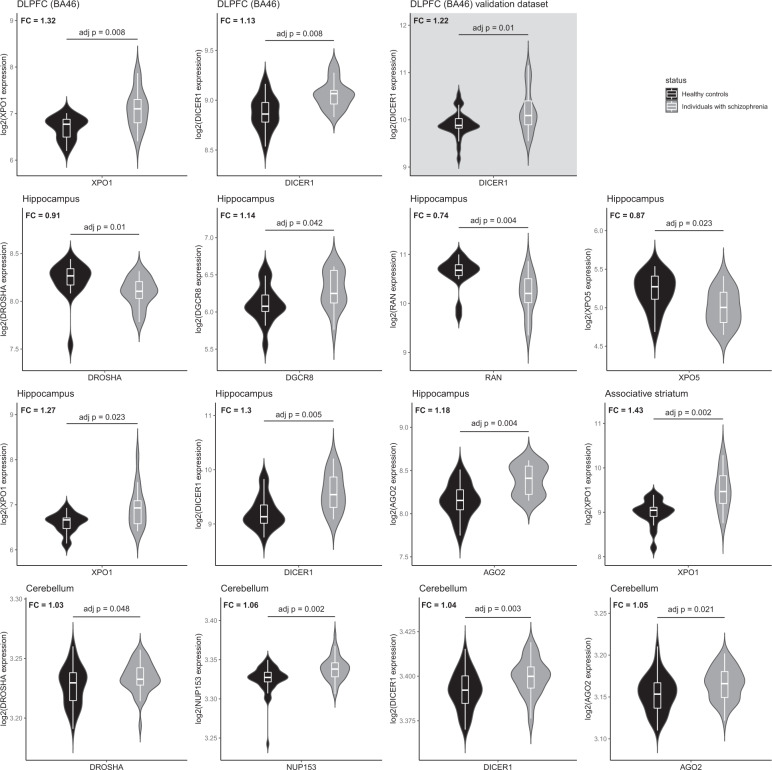
Differentially expressed genes in the postmortem brain samples of individuals with schizophrenia relative to healthy controls. Shown are violin plots displaying the expression distribution of each gene with overlaid box plots. The violin plots are filled in black for healthy controls and grey for individuals with schizophrenia. Results obtained from primary and validation datasets are presented on a white and grey background, respectively. For each gene, fold change (FC) represents the expression of the target gene in individuals with schizophrenia relative to that in healthy controls. DLPFC dorsolateral prefrontal cortex, BA Brodmann area. Adjusted *p*-values set at 0.05.

Compared to HC, we observed distinct altered expression patterns of the miRNA BM coding genes in the brain tissues of SZ individuals with the exception of the anterior prefrontal (BA10), parietal and superior temporal (BA22) cortices in which no alterations were found. SZ individuals exhibited a set of genes significantly upregulated in the DLPFC (BA46) (XPO1 and DICER1), the associative striatum (XPO1) and in the cerebellum (DROSHA, NUP153, DICER1, and AGO2). Moreover, altered transcription of several of the candidate genes was identified in the hippocampus (DROSHA, DGCR8, RAN, XPO5, XPO1, DICER1, and AGO2). In the validation dataset derived from DLPFC (BA46) samples, we replicated DICER1 overexpression in SZ individuals vs. HC. The detailed results are provided in Fig. 2 and Supplementary Table 4.

### Differential expression analysis in the peripheral tissues

**Fig. 3:**
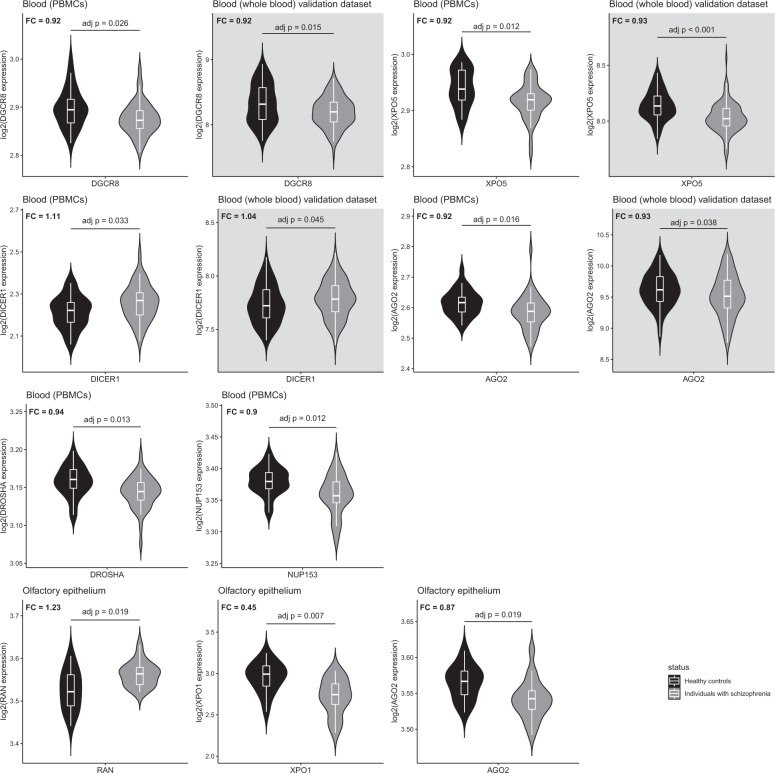
Differentially expressed genes in the peripheral tissues samples of individuals with schizophrenia relative to healthy controls. Shown are violin plots displaying the expression distribution of each gene with overlaid box plots. The violin plots are filled in black for healthy controls and grey for individuals with schizophrenia. Results obtained from primary and validation datasets are presented on a white and grey background, respectively. For each gene, fold change (FC) represents the expression of the target gene in individuals with schizophrenia relative to that in healthy controls. PBMCs peripheral mononuclear cells. Adjusted *p*-values set at 0.05.

Compared to HC, distinct altered transcription patterns of the miRNA BM coding genes were identified in the peripheral tissues of SZ individuals. SZ individuals exhibited a set of differentially expressed genes in the PBMCs (DROSHA, DGCR8, XPO5, NUP153, DICER1, and AGO2) and in the olfactory epithelium (RAN, XPO1, and AGO2). In contrast, no changes were found in the skin fibroblasts. In the validation dataset derived from whole blood samples, we replicated DICER1 overexpression and DGCR8, XPO5, AGO2 decreased expression in SZ individuals vs. HC. The detailed results are provided in Fig. 3 and Supplementary Table 5.

## Discussion

Compared to HC, we observed altered expression patterns of the miRNA BM coding genes both in brain and peripheral tissues of SZ individuals. These patterns were distinct between the different tissues. All the original studies from which are derived the included datasets used a genome-wide approach. As a principle, the expression of all genes was analyzed, including those involved in the miRNA BM. However, while some original studies have reported differential expression of these genes in their supplementary data^36,42^, those results were not discussed in the original experiments. The present study re-analyzed the original data using a candidate-gene approach, allowing us to extend previous knowledge on schizophrenia-associated microRNA BM dysregulation to other brain regions and peripheral tissues.

First, we report distinct altered transcriptional patterns of the miRNA BM coding genes in four brain regions (the DLPFC, associative striatum, hippocampus, and cerebellum) of SZ individuals vs. HC. Notably, heterogeneous results have been identified both at the gene-level (some miRNA BM genes undergo significant expression changes whereas others show no alteration) and at the brain region-level (the altered transcriptional patterns differ from one brain region to another). Such distinct abnormal transcription patterns have been previously reported in two different brain regions, namely the DLPFC (including BA9 and BA46) and the superior temporal gyrus (BA22)^30,31^. The spatial heterogeneity of the observed transcriptional alterations may contribute to the distinct abnormal expression patterns of miRNA^28,30,31,54–60^ and subsequently, to the heterogeneous abnormal mRNA levels^7,8,61^ (through miRNA-induced mRNA degradation) and protein levels^62–64^ (through the interference of mRNA translation) observed in various brain regions of SZ individuals.

In the DLPFC (BA46), associative striatum and cerebellum of SZ individuals vs. HC, all the explored miRNA BM genes whose expression was altered were overexpressed, suggesting a heightened miRNA production in these brain regions. Our results are consistent with the increased expression of miRNAs previously reported in the DLPFC (BA46) of SZ individuals^31^. In the DLPFC, overexpression of miRNAs could account for the emergence of SZ during the brain maturation period of late adolescence. Indeed, in the DLPFC, global miRNA expression physiologically displays a reduction from adolescence, with a predominant inflexion point at 17 years^65^. In adolescence, overexpression of the miRNA BM may thus constitute a disruption of the posttranscriptional regulatory environment. In adulthood, persistent overexpression of miRNA BM genes may reflect immaturity of the brain tissue which is in accordance with the report that miRNAs normally enriched in infants tend to be upregulated in SZ individuals^66^.

To our knowledge, it is the first time that an overexpression of the miRNA biogenesis genes is reported in the associative striatum and the cerebellum of SZ individuals. Since, none of the available studies exploring the cerebral expression of miRNA in SZ individuals have examined cerebellum or striatum tissues^28,30,31,54–60^, more studies are needed to determine if the herein reported overexpression of the miRNA BM genes is associated with a global increase in miRNA expression in these areas. In this regard, high quality brain collections are compulsory considering the limited stability and short half-life of specific brain-enriched miRNAs^67^. We also report an altered transcriptional pattern of miRNA BM coding genes in the hippocampus of SZ individuals. While some miRNA BM genes were overexpressed, others were underexpressed. The complexity of this altered transcriptional pattern does not allow for a clear hypothesis on the overall level of miRNA in this structure.

While previous studies observed aberrant transcription of the miRNA BM in the DLPFC BA46 and BA9^30,31^, our results expand this epigenetic disruption to subcortical structures and to the cerebellum. This is in accordance with the central role of these areas in SZ pathogenesis^68^. On the one hand, all these regions exert crucial roles in cognition processes^46,69–71^, and are considered as key regions in the pathophysiology of schizophrenia^44–47^. On the other hand, there is now increasing evidence that miRNAs play an important role in various aspects of synaptic plasticity^21^, and that the dynamic changes in miRNA levels regulate the expression of genes involved in cognitive processes^72^. Disruption of the miRNA BM in brain structures involved in cognitive functions may thus participate to the cognitive impairment associated with schizophrenic disorders^66,73^.

In the blood compartment and olfactory epithelium of SZ individuals vs. HC, we observed two distinct altered transcriptional patterns characterized by an overall underexpression of the candidate genes (except for the overexpression of DICER1 and RAN in the blood compartment and olfactory epithelium, respectively). In contrast, no changes were found in the skin fibroblasts.

Although skin fibroblasts are considered as a promising surrogate system for the study of SZ pathogenesis^74^, our results suggest that they may not be affected by transcriptional alterations of the miRNA BM. In PBMCs, whole blood and olfactory epithelium of SZ individuals the aberrant transcription of the candidate genes is consistent with the previously reported altered expression of a wide range of miRNAs in peripheral blood^75–77^, and olfactory epithelium^78^ of SZ individuals. However, in the present study, the transcriptional alterations observed in peripheral tissues did not reflect those identified in the brain tissues of SZ individuals (with the exception of DICER1 overexpression in the blood compartment). Such discrepancies were expected since (i) peripheral blood and olfactory epithelium have specific expression profiles, distinct from that of the brain tissue, thus, they can’t be perfect surrogates for gene expression in the brain^79,80^, (ii) brain and peripheral tissues samples used in the present study were not derived from the same subjects and (iii) brain and peripheral tissues of SZ individuals might be affected by different alterations^79^. Nevertheless, such discrepancies does not preclude the possibility that transcriptional alterations observed in peripheral tissues could be used as clinical biomarkers of schizophrenia^18,74–76,78,79^. More studies in peripheral tissues are needed to identify effective sources of biomarkers for SZ.

Among individual genes whose expression was altered in the present study, a significant finding was the increased expression of DICER1 in the DLPFC (BA46), hippocampus, cerebellum and PBMCs of SZ individuals compared to HC. Moreover, in the blood compartment, we replicated DICER1 overexpression in the whole blood of SZ individuals vs. HC. It is noteworthy that DICER1 expression is age-dependent with a significant increase from young adulthood onward^65^. However, since there was no significant difference between the mean age of SZ individuals and HC in the different datasets, it is unlikely that age explains our results.

In the DLPFC (BA46), our results are consistent with two previous studies reporting a significant upregulation of DICER1 in the DLPFC (BA46 and BA9) of SZ individuals from an independent collection of postmortem brain samples^30,31^. On the one hand, DICER1 is essential for neural and synaptic plasticity^81^. Moreover, DICER1 codes for the Dicer enzyme, which is involved in neural cell differentiation^50^ and immune cell regulation^82^. On the other hand, the DICER1 overexpression observed in the present study is likely associated with a global increase in miRNA expression as reported in the DLPFC of SZ individuals^31^. Such an association between an upregulation of Dicer and a global increase in miRNA expression has been previously shown in tumorous cells^83^. Altogether, these results suggest that DICER1 overexpression in brain tissues of SZ individuals may be associated with an increased miRNA production, leading to extensive detrimental consequences on synaptic and neural plasticity as well as on immune cell function. DICER1 overexpression may constitute a key mechanism in the SZ pathogenesis. The association between genetic polymorphisms in DICER1 and increased risk to develop SZ sustains this hypothesis^27,28,84,85^.

Remarkably, in the blood compartment as well, we report DICER1 overexpression in the PBMCs and whole blood of SZ individuals vs. HC. These results are consistent with two previous peripheral studies. A genome-wide significant overexpression of DICER1 was identified in the lymphoblastoid cell lines (LCL) of SZ individuals vs. HC^86^. DICER1 overexpression was also reported in the whole blood of treatment-resistant SZ individuals vs. HC^11^. In the whole blood of individuals with first episode psychosis (FEP), DICER1 overexpression may depend on the treatment status and IL-6 peripheral levels^87–89^. DICER1 overexpression in the blood compartment may thus be a clinically useful peripheral biomarker of SZ. More studies are needed to assess the interaction between antipsychotic treatment and IL-6/DICER1 blood expression levels.

In SZ individuals, we observed an altered expression of DROSHA and DGCR8 genes encoding proteins involved in the nuclear maturation step of miRNAs.

At the brain level, the decreased hippocampal expression of DROSHA contrasted with its upregulation in the cerebellum. These results are consistent with and extend those of two previous studies who reported DROSHA overexpression in the DLPFC (BA9) of SZ individuals, while no alteration was observed in the DLPFC (BA46) and superior temporal gyrus (BA22)^30,31^. The DGCR8 hippocampal overexpression observed in the present experiment is consistent with the increased expression of DGCR8 reported in the DLPFC (BA9) and superior temporal cortex (BA22) of SZ individuals^30^. However, we did not confirm DGCR8 overexpression in the superior temporal cortex (BA22) of SZ individuals. These discrepant results may be due to the heterogeneity of the demographic and clinical characteristics of the human samples: age, illness duration, nature, and/or severity of symptomatology may influence the miRNA biogenesis system and thus constitutes confounding factors. While illness duration and clinical features were not available in both studies, the population presently studied was older than that studied by Beveridge et al.^30^ (mean age ± SD; present study: SZ individuals: 72.2 ± 16.9 years/control subjects: 67.7 ± 22.2 years; Beveridge et al. study: SZ individuals: 52.7 ± 11.7 years/control subjects: 53.2 ± 11.4 years). Altogether these results suggest that the nuclear processing step of miRNAs biogenesis may be altered in various brain regions of SZ individuals. Interestingly, overexpression of DROSHA may exert detrimental effects on the miRNA production through the destabilization of DGCR8 mRNA by Drosha enzyme^50^. Moreover, DGCR8 is located within the 22q11.2 region which is prone to spontaneous structural variation. 22q11.2 microdeletion (leading to decreased expression of DGCR8) is the strongest known genetic risk factor for SZ disorders^90^. 22q11.2 microduplication (leading to overexpression of DGCR8) has also been recently reported in SZ individuals^91^ and shown to increase risk for intellectual disability and autism spectrum disorder^92,93^. DGCR8 abnormal expression may thus constitute a nonspecific risk factor for neurodevelopmental disorders.

At the peripheral level, we observed a decreased expression of DROSHA and DGCR8 in the PBMCs of SZ individuals, suggesting a reduction in miRNAs nuclear processing in the blood compartment. However, when considering previous results from whole blood samples of FEP individuals vs. HC, mixed results have been reported regarding DROSHA and DGCR8 expression^87–89^. Such discrepancies between PBMCs and whole blood are expected since those two tissues have distinct expression profiles^94^. However, additional factors such as illness duration^87–89^, antipsychotic status^89^, peripheral cytokine levels^88^, or antipsychotic-IL-6 interaction^88^ may be involved in the mixed results observed in the blood compartment.

Interestingly, we report for the first time XPO1 altered expression in SZ individuals compared to HC. In the present study, XPO1 was overexpressed in the DLPFC (BA46), the associative striatum and the hippocampus, whereas it was downregulated in the olfactory epithelium. This is coherent with previous results reporting XPO1 as a contributing factor to neurodevelopmental disorders^95^.

Apart from the canonical biogenesis pathway, various alternative factors have been implicated in miRNA biogenesis^50^. Notably, while the majority of premiRNAs are exported to the cytoplasm through the canonical pathway (involving exportin 5 and RAN nuclear protein, encoded by XPO5 and RAN, respectively), the exportin 1 protein (encoded by XPO1), involved in a noncanonical pathway, can export specific miRNAs to the cytoplasm^50,51^. Moreover, during cellular quiescence, the canonical miRNA biogenesis pathway is downregulated and specific miRNAs are generated by an alternative exportin 1-dependent pathway^96^. In the DLPFC (BA46), associative striatum and hippocampus, our results raise for the first time the intriguing question of whether schizophrenia is associated with a shift towards the activation of a noncanonical miRNA biogenesis pathways in cerebral tissues^97^. Consistently, in the hippocampus of SZ individuals, XPO1 overexpression contrasted with the decreased expression of the canonical miRNA biogenesis genes XPO5 and RAN. Intriguingly, we observed opposite transcriptional abnormalities in the olfactory epithelium (downregulation of XPO1 and overexpression of RAN) suggesting that the putative shift towards a noncanonical pathway may be restricted to cerebral tissues of SZ individuals.

In the present study, AGO2 expression was altered in opposite directions between brain and peripheral tissues of SZ individuals: it was overexpressed in the hippocampus and cerebellum, whereas its expression was decreased in the PBMCs, whole blood, and olfactory epithelium. Our results replicate and extend the decreased expression of AGO2 previously reported in the PBMCs of SZ individuals^32^. Further research is needed to determine if peripheral downregulation of AGO2 expression could represent a biomarker of SZ.

Recently, AGO2 has been functionally linked to deleterious stress exposure. Indeed, Bam et al. reported a decreased expression of AGO2 in the PBMCs of war veterans suffering from PTSD^98^. In this latter study, AGO2 decreased expression was associated with the underexpression of numerous miRNAs targeting proinflammatory genes and lead to PTSD-associated chronic peripheral inflammation. At the brain level, animal models have shown that specific miRNAs associate with Ago2 protein in the amygdala following chronic stress^99,100^. Altogether, these results suggest that AGO2 may be involved in stress response mechanisms and that its altered expression may be associated to detrimental stress exposures. In the present study, traumatic events were not provided, thus we could not explore a possible link between AGO2 altered expression and exposure to stressful events. More studies are needed to explore the impact of stress on the miRNA BM.

### Limits

Interpretation of the present results is affected by limiting factors. First, brain and peripheral tissues were obtained from eight different and modest-sized SZ samples. PBMCs were collected from rather young men (mean age ± SD: 23.02 ± 4.03 for the controls and 23.90 ± 4.08 for the SZ individuals), whereas brain tissues were obtained from older men and women (Table 1). Therefore, the gene expression changes identified in the cerebral tissues may reflect the influence of age, while those identified in the PBMCs may be gender specific. However, previous studies investigating miRNA BM expression in the cerebral cortex did not find any significant correlation between gender and gene expression levels^30,31,65^. In contrast, Beveridge et al. reported that DICER1 (but not DROSHA or DGCR8) expression increased with age^65^. However, since we did not find any significant difference in age between control subjects and SZ individuals in the different datasets used in the present study (Tables 1 and 2), it is unlikely that age explain our results. Second, factors inherent in postmortem brain studies, and beyond the investigator’s control, might have influenced our results. (i) Treatment data were not provided for the GEO datasets. Thus, a potential impact of antipsychotic treatment on candidate gene expression could not be ruled out. In individuals with FEP, baseline circulating IL-6 levels may modulated the gene expression response of DICER1 and DROSHA to treatment with risperidone^88^. Further studies are needed to replicate these results. (ii) The other concern is the effect of brain pH on our results since we observed a significant difference in brain pH between control and SZ patient groups in the anterior PFC (BA10) and superior temporal cortex. However, the main results discussed in the present study do not derive from these samples.

This study provides evidence of a widespread transcriptional disruption of the miRNA BM both in brain and peripheral tissues of SZ individuals. In the DLPFC (BA46), associative striatum and cerebellum of SZ individuals, we observed an overexpression pattern of the candidate genes suggesting a heightened miRNA production in these brain regions. In SZ individuals, the transcriptional abnormalities were further extended to the hippocampus. These aberrant transcription patterns may contribute to SZ pathogenesis. Moreover, we observed distinct transcriptional abnormalities of the miRNA BM in peripheral tissues suggesting that SZ is a systemic disease. In SZ individuals, we finally reported a DICER1 overexpression in the hippocampus, cerebellum, and DLPFC (BA46) (consistently with two previous reports). We furthermore reported a congruent overexpression of DICER1 in the peripheral blood of two independent samples of SZ individuals. These latter results replicate two previous observations and suggest that it may represent a clinically useful peripheral marker.

## Supplementary information

Supplementary Tables 1-5

## Data Availability

The data that support the findings of this study are openly available in Gene Expression Omnibus (NCBI) at https://www.ncbi.nlm.nih.gov/geo/, reference numbers, GSE17612, GEO21138, GSE21935, GSE27383, GSE35974, GSE35977, GEO38484, GSE53987, GSE62333, GSE73129.
