# Supplementary material for: Widespread transcriptional disruption of the microRNA biogenesis machinery in brain and peripheral tissues of individuals with schizophrenia
Source: Transl Psychiatry. 2020 Nov 4;10:376. doi: 10.1038/s41398-020-01052-5 (PMC7642431; doi:10.1038/s41398-020-01052-5)
Supplement: Supplementary file 1 — Supplementary Tables 1-5 [file 41398_2020_1052_MOESM1_ESM.pdf]

Supplementary Table 1 Description of the original studies from which the primary datasets were obtained

Supplementary Table 2 Description of the original studies from which the validation datasets were obtained

Supplementary Table 3 Descriptive Statistics of log2(candidate gene expression) values in brain and peripheral tissues

Supplementary Table 4 Genes with altered expression in the postmortem brain samples of individuals with schizophrenia relative to healthy controls

Supplementary Table 5 Genes with altered expression in the peripheral tissue samples of individuals with schizophrenia relative to healthy controls

Supplementary Table 1 Description of the original studies from which the primary datasets were obtained

<table>
<tr><th>GEO Accession Number</th><th>Tissue</th><th>Region / Cell Type</th><th>Microarray</th><th>Individuals with schizophrenia</th><th>Control subjects</th><th colspan="2">Description of the original study</th></tr>
<tr><td>GSE 53987</td><td>Brain</td><td>DLPFC (BA46)</td><td>HG-U133_Plus_2</td><td>15</td><td>19</td><td rowspan="3">Sample collection:

Consent / Ethics:

SZ individuals:

Healthy controls:

Original study:

Objective:

Methods:

Main results:</td><td rowspan="3">University of Pittsburgh brain bank

Brain specimens were obtained during autopsies after consent for donation was obtained from the next-of-kin.
All procedures were approved by the University of Pittsburgh Committee for the Oversight of Research and Clinical Trials Involving the Dead and the Institutional Review Board for Biomedical Research

Diagnoses were made by an independent committee of experienced research clinicians, using DSM-IV criteria and based on the results of structured interviews conducted with family members and review of medical records

The absence of psychiatric diagnoses was confirmed using an identical approach. The healthy controls were free of any neurological or psychiatric illness during their life course

Lanz et al., Translational Psychiatry, 2019 (PMID: 31123247)

To evaluate shared transcriptional alterations across connected brain regions in SZ, bipolar disorder (BD), major depressive disorder (MDD) individuals, or HC

Genome-wide expression was obtained from postmortem dorsolateral prefrontal cortex (DLPFC), hippocampus, and associative striatum from 19 well-matched tetrads of subjects with SCZ, bipolar disorder (BD), major depressive disorder (MDD), or healthy controls

SZ individuals showed a substantial burden of differentially expressed genes across all examined brain regions with the greatest effects in hippocampus, whereas BD and MDD showed less robust alterations. Pathway analysis of transcriptional profiles compared across diagnoses demonstrated commonly enriched pathways between all three disorders in hippocampus, significant overlap between SCZ and BD in DLPFC, but no significant overlap of enriched pathways between disorders in striatum. SZ individuals showed increased expression of transcripts associated with inflammation across all brain regions examined, which was not evident in BD or MDD individuals</td></tr>
<tr><td>GSE 53987</td><td>Brain</td><td>Hippocampus</td><td>HG-U133_Plus_2</td><td>15</td><td>18</td></tr>
<tr><td>GSE 53987</td><td>Brain</td><td>Associative striatum</td><td>HG-U133_Plus_2</td><td>18</td><td>18</td></tr>
<tr><td>GSE 35977</td><td>Brain</td><td>Parietal cortex</td><td>HuGene-1_0-st</td><td>51</td><td>50</td><td rowspan="2">Sample collection:

Consent / Ethics:

SZ individuals:

Healthy controls:

Exclusion criteria:

Original study:

Objective:

Methods:

Main results:</td><td rowspan="2">Stanley Medical Research Institute's Neuropathology Consortium and Array Collections

Specimens were collected with informed consent from next-of-kin

Diagnoses were made by two senior psychiatrists, using DSM-IV criteria and based on medical records and, when necessary, telephone interviews with family members

Diagnoses of unaffected controls were based on structured interviews by a senior psychiatrist with family member(s) to rule out Axis I diagnoses.

Individuals over age 65 were excluded

Chen et al., Molecular Psychiatry, 2013 (PMID: 23147385)

To identify schizophrenia-associated gene-expression networks in the parietal cortex and cerebellum of SZ individuals and HC. To test whether the gene modules perturbed in SZ were similarly perturbed in BD

Genome-wide expression data was used to construct gene expression networks and identify gene co-expression modules within the networks. The modules were tested for association with schizophrenia. Identified SZ-associated modules were tested for association with BD

Two modules were differentially expressed in SZ individuals versus HC. One, upregulated in cerebral cortex, was enriched with neuron differentiation and neuron development genes, as well as disease genome-wide association study genetic signals; the second, altered in cerebral cortex and cerebellum, was enriched with genes involved in neuron protection function. The findings were preserved in five expression datasets, including sets from three brain regions, from a different microarray platform, and from BD individuals</td></tr>
<tr><td>GSE 35974</td><td>Brain</td><td>Cerebellum</td><td>HuGene-1_0-st</td><td>14</td><td>14</td></tr>
</table>

Supplementary Table 1 (continued)

<table>
<tr><th>GEO Accession Number</th><th>Tissue</th><th>Region / Cell Type</th><th>Microarray</th><th>Individuals with schizophrenia</th><th>Control subjects</th><th colspan="3">Description of the original study</th></tr>
<tr><td rowspan="5">GSE 17612</td><td rowspan="5">Brain</td><td rowspan="5">Anterior PFC (BA10)</td><td rowspan="5">HG-U133_Plus_2</td><td rowspan="5">28</td><td rowspan="5">23</td><td>Sample collection:</td><td colspan="2">Tissue collection of the Charing Cross Hospital, Imperial College London, UK</td></tr>
<tr><td>Consent / Ethics:</td><td colspan="2">All patients with the agreement of their nearest relative or authorized representative, have given written informed consent for use of tissue obtained post-mortem for research. The control group were tissue donors for research from the community. Procedures have been approved by the West London Mental Health Ethical Research Committee and complies with the conditions of the Research Governance Office of the Imperial College of Science, Technology and Medicine Clinical Research Office</td></tr>
<tr><td>SZ individuals:</td><td colspan="2">All patients met DSM-III diagnostic criteria for chronic residual schizophrenia with pronounced negative symptoms alongside attenuated positive symptoms and intellectual dysfunction. All patients had been treated with neuroleptic drugs except one patient who was neuroleptic naive at death</td></tr>
<tr><td>Healthy controls:</td><td colspan="2">Mentally normal individuals from the community</td></tr>
<tr><td>Exclusion criteria:</td><td colspan="2">Alzheimer’s disease, Parkinson’s disease or multiple sclerosis were excluded</td></tr>
<tr><td rowspan="4">GSE 21935</td><td rowspan="4">Brain</td><td rowspan="4">Superior temporal cortex (BA22)</td><td rowspan="4">HG-U133_Plus_2</td><td rowspan="4">23</td><td rowspan="4">19</td><td>Original studies:</td><td>Maycox et al., Mol Psychiatry, 2009 (PMID: 19255580)</td><td>Barnes et al., J Neurosci Res, 2011 (PMID: 21538462)</td></tr>
<tr><td>Objective:</td><td>To identify differentially expressed genes in anterior prefrontal cortex (BA 10) from SZ individuals and HC</td><td>Gene ontology pathway enrichment analysis in BA22 and BA10 from SZ individuals and HC</td></tr>
<tr><td>Methods:</td><td>Genome-wide expression in post-mortem brain tissue from anterior prefrontal cortex (BA 10) was compared between 28 SZ individuals and 23 HC. Results were then compared to those from an independent prefrontal cortex dataset obtained from SZ individuals and HC</td><td>Genome-wide expression was determined in the post-mortem BA22 region of 23 SZ individuals and 19 HC and compared with genome-wide expression of BA10 from the same subjects. Gene ontology pathway enrichment analysis was carried out in each region</td></tr>
<tr><td>Main results:</td><td>51 gene expression changes were common between the two schizophrenia cohorts, and 49 showed the same direction of disease-associated regulation. Changes were observed in gene sets associated with synaptic vesicle recycling, transmitter release and cytoskeletal dynamics</td><td>In BA22 region, the highest enrichment was observed in processes mediating cell adhesion, synaptic contact, cytoskeletal remodeling, and apoptosis. In BA10 region, the strongest changes were observed in reproductive signaling, tissue remodeling, and cell differentiation. In schizophrenia, many pathways underpinning synaptic plasticity are disrupted in both BA10 and BA22</td></tr>
<tr><td rowspan="9">GSE73129</td><td rowspan="9">Olfactory Epithelium</td><td rowspan="9">Neural layers</td><td rowspan="9">HG-U133_Plus_2</td><td rowspan="9">19</td><td rowspan="9">19</td><td>Sample collection:</td><td colspan="2">Johns Hopkins Schizophrenia Center, Baltimore, MD, USA</td></tr>
<tr><td>Consent / Ethics:</td><td colspan="2">The study was conducted under approval of the Johns Hopkins Institutional Review Board. All the subjects gave written consent for their participation</td></tr>
<tr><td>SZ individuals:</td><td colspan="2">Patients with chronic SZ were recruited. Diagnosis was determined according to the DSM-IV using the Structured Clinical Interview for DSM-IV Axis I Disorders-Clinician Version</td></tr>
<tr><td>Healthy controls:</td><td colspan="2">Normal control subjects were recruited from the general population. All subjects were administered the Structured Clinical Interview for DSM-IV Axis I Disorders-Clinician Version</td></tr>
<tr><td>Exclusion criteria:</td><td colspan="2">Subjects were excluded from the study if they had a history of traumatic brain injury with loss of consciousness for 41h, a history of drug abuse within 6 months of the study, a history of drug dependence within 12 months of the study or a history of untreated major medical illnesses</td></tr>
<tr><td>Original study:</td><td colspan="2">Horiuchi et al., Translational Psychiatry, 2016 (PMID: 27727244)</td></tr>
<tr><td>Objective:</td><td colspan="2">To identify differentially expressed genes and correlation between those genes and neuropsychological traits</td></tr>
<tr><td>Methods:</td><td colspan="2">Genome-wide expression study in the olfactory neuronal layers of SZ individuals compared to HC, in parallel with systematic neuropsychological assessments on the same participants</td></tr>
<tr><td>Main results:</td><td colspan="2">2,574 transcripts were differentially expressed in SZ individuals. 17 transcripts correlated with SZ neuropsychology scores. Differentially regulated genes in SZ were filtered based on correlation with neuropsychological traits leading to identification of SMAD 5 gene. RT-PCR analysis supported downregulation of the SMAD pathway in SZ</td></tr>
</table>

Supplementary Table 1 (continued)

GEO Accession Number	Tissue	Region / Cell Type	Microarray	Individuals with schizophrenia	Control subjects	Description of the original study	
GSE 27383	Blood	PBMCs	HG-U133_Plus_2	43	29	**Sample collection:**	Erasmus University Medical Center (EMC), Rotterdam, The Netherlands
						**Consent / Ethics:**	All subjects provided written informed consent after complete description of the study. For those patients who were too disturbed to provide consent, consent was initially given by a first-degree relative and final written consent was sought within six weeks from the patients themselves. This study was approved by the Erasmus University Medical Center Institutional Review Board and was conducted according to the declaration of Helsinki
						**SZ individuals:**	Eligible for inclusion were male, stabilized or acutely psychotic, patients diagnosed with schizophrenia or schizophreniform disorder according to DSM IV criteria after a Comprehensive Assessment of Symptoms and History interview (CASH) and by consensus between two senior psychiatrists. Additional criteria were recent onset (defined as duration of illness <5 years) and age (>15 and <36 years)
						**Healthy controls:**	Age-matched controls were recruited from the students and staff of the EMC medical school and hospital
						**Exclusion criteria:**	For SZ individuals and HC, exclusion criteria were defined as follows: presence of any somatic or neurological disorders and abuse of heroin, cocaine, or alcohol. Cannabis abuse was not an exclusion criterion. Concomitant use of mood-stabilizers and/or antidepressants was an exclusion criterion. For HC, the presence of psychiatric disorders in first-degree relatives was also an exclusion criterion
						**Original study:**	van Beveren *et al.*, PLoS ONE, 2012 (PMID: 22393424)
						**Objective:**	To examine PBMC expression levels of AKT1 in SZ individuals versus HC, and to examine whether functional biological processes in which AKT1 plays an important role are deregulated in SZ individuals
						**Methods:**	Genome-wide expression study in the PBMCs of SZ individuals and HC. Functional analysis of biological processes in which AKT1 gene is involved
						**Main results:**	PBMC expression of AKT1 was significantly decreased in SZ individuals. 1224 genes were differentially expressed between SZ individuals and controls. Deregulated canonical pathways were involved in cellular processes: immune system, cell adhesion and neuronal guidance, neurotrophins and (neural) growth factors, oxidative stress and glucose metabolism, apoptosis and cell-cycle regulation. Many of these processes are associated with AKT1
GSE 62333	Skin	Fibroblasts	HuGene-1_1-st	20	20	**Sample collection:**	Samples from Brescia and Verona, Italy
						**Consent / Ethics:**	The project was approved by the local ethics committee. Written informed consent was obtained from the patients and controls. In the case of patients with a compromised ability to provide authorization, informed consent was signed by the legally authorized representative
						**SZ individuals:**	All SZ individuals satisfied the DSM-IV criteria for SCZ. Diagnoses were confirmed using the Structured Clinical Interview for DSM-IV Axis I Disorders (SCID-I) diagnostic scale
						**Healthy controls:**	Unrelated healthy volunteers were screened for DSM-IV Axis I disorders by expert psychologists using the Mini-International Neuropsychiatric Interview (M.I.N.I.). Only healthy volunteers without a history of drug or alcohol abuse or dependence and without a personal or first-degree family history of psychiatric disorders were enrolled in the study
						**Exclusion criteria:**	For SZ individuals and HC, exclusion criteria were defined as follows: mental retardation or cognitive disorder; serious somatic illnesses; uncorrected hypothyroidism or hyperthyroidism; age <18 and >70 years; metabolic disorders (diabetes); specific dermal diseases (e.g., dermal cancer or psoriasis). For HC, the presence of a history of drug, alcohol abuse or dependence, the presence of psychiatric disorders in first-degree relatives were additional exclusion criteria
						**Original study:**	Cattane *et al.*, PLoS ONE, 2015 (PMID: 25658856)
						**Objective:**	Differential expression analysis in skin fibroblasts of SZ individuals and HC
						**Methods:**	Genome-wide expression study comparing skin fibroblast transcriptomic profiles from 20 SZ individuals and 20 HC
						**Main results:**	Six genes (JUN, HIST2H2BE, FOSB, FOS, EGR1, TCF4) were strongly and significantly upregulated at the genome-wide level and confirmed by RT-PCR in SC individuals compared to HC

DLPFC dorsolateral prefrontal cortex, *BA* Brodmann area, *PFC* prefrontal cortex, *PBMCs* peripheral blood mononuclear cells, *SZ* schizophrenia, *BD* bipolar disorder, *MDD* major depressive disorder, *HC* healthy controls

Supplementary Table 2 Description of the original studies from which the validation datasets were obtained

GEO Accession Number	Tissue	Region / Cell Type	Microarray	Individuals with schizophrenia	Control subjects	Description of the original study	
GSE 21138	Brain	DLPFC (BA46)	HG-U133_Plus_2	30	29	**Sample collection:**	Victorian Brain Bank Network
						**Consent / Ethics:**	The study was conducted under approval of the Ethics Committee of the Victorian Institute of Forensic Medicine and the North Western Mental Health Program Behavioral and Psychiatric Research and Ethics Committee
						**SZ individualss:**	Psychiatric diagnoses of schizophrenia were made according to DSM-IV criteria by consensus between two senior psychiatrists and a psychologist following extensive case history review using the Diagnostic Instrument for Brain Studies (DIBS). All subjects were treated with typical antipsychotic drugs. Death by suicide recorded
						**Healthy controls:**	Age- and sex-matched unaffected controls
						**Original study:**	Narayan *et al.*, Brain Research, 2008 (PMID: 18778695)
						**Objective:**	To identify differentially expressed genes depending on the stage of illness, in the DLPFC of 30 SZ individuals compared to 29 HC
						**Methods:**	Subjects were divided into three cohorts to represent different stages of illness: SZ individuals very close to initial diagnosis (≤ 4 years duration of illness (DOI)), chronic schizophrenics who had long-term DOI (> 28 years of illness), SZ individuals with intermediate ranges of DOI (7–18 years of illness). Genome-wide expression profiling in each of the 3 sub-cohorts
						**Main results:**	The greatest number and magnitude of gene expression differences were detected in SZ individuals with short-term illness. Each defined stage of illness was associated with dysfunction in both distinct, as well as overlapping systems. Short-term illness was associated with disruptions in gene transcription, metal ion binding, RNA processing and vesicle-mediated transport. Long-term illness was associated with inflammation, stimulus-response and immune functions
GSE 38484	Blood	Whole blood	HumanHT-12 V3.0	106	96	**Sample collection:**	Department of Psychiatry, University Medical Center, Utrecht, The Netherlands / Parnassia PsychoMedical Center, The Netherlands / Center for Neuropsychiatric Schizophrenia Research, Psychiatric Center Glostrup, Denmark
						**Consent / Ethics:**	The study was approved by Medical Research Ethics Committee (METC) of the University Medical Center Utrecht, The Netherlands and the Committees on Biomedical Research Ethics for the Capital Region of Denmark. All participants gave written informed consent
						**SZ individuals:**	Psychiatric diagnoses of schizophrenia were made according to DSM-IV-TR criteria by trained clinicians using Standardized Psychiatric interviews either The Comprehensive Assessment of Symptoms and History (CASH) or the Composite international diagnostic interview (CIDI)
						**Healthy controls:**	Unaffected controls
						**Original study:**	de Jong *et al.*, PLoS ONE, 2012 (PMID: 22761806)
						**Objective:**	To identify schizophrenia-associated gene co-expression modules in the whole blood of SZ individuals compared to HC
						**Methods:**	Genome-wide expression profiling from whole blood of 106 SZ individuals and 96 HC. Schizophrenia-associated gene co-expression modules
						**Main results:**	Identification of 12 large gene co-expression modules associated with schizophrenia. Two of the schizophrenia-associated modules were replicated in an independent second dataset involving antipsychotic-free SZ individuals and HC. One of these schizophrenia-associated modules is significantly enriched with brain-expressed genes and with genetic risk variants for schizophrenia, the hub gene in this module (ABCF1) is located in and regulated by the MHC-complex

DLPFC dorsolateral prefrontal cortex, *BA* Brodmann area, *SZ* schizophrenia, *HC* healthy controls

Supplementary Table 3 Descriptive Statistics of log2(candidate gene expression) values in brain and peripheral tissues

DLPFC (BA46)

	log2(DROSHA)		log2(DGCR8)		log2(RAN)		log2(XPO5)		log2(NUP153)		log2(XPO1)		log2(DICER1)		log2(AGO2)	
	Ctrl	Sz	Ctrl	Sz	Crtl	Sz	Ctrl	Sz	Ctrl	Sz	Ctrl	Sz	Ctrl	Sz	Ctrl	Sz
**Valid**	19	15	19	15	19	15	19	15	19	15	19	15	19	15	19	15
**Missing**	0	0	0	0	0	0	0	0	0	0	0	0	0	0	0	0
**Mean**	8.776	8.794	6.086	6.096	11.230	11.207	5.533	5.448	8.291	8.344	6.696	7.097	8.873	9.055	4.333	4.585
**Std. Deviation**	0.111	0.081	0.166	0.148	0.218	0.180	0.194	0.166	0.108	0.094	0.239	0.414	0.167	0.144	0.222	0.359
**Minimum**	8.525	8.605	5.773	5.846	10.476	10.783	5.063	5.230	8.063	8.097	6.199	6.406	8.535	8.831	3.919	4.142
**Maximum**	8.939	8.876	6.443	6.383	11.451	11.394	5.880	5.796	8.520	8.460	7.009	7.868	9.164	9.357	4.699	5.206

Anterior PFC (BA10)

	log2(DROSHA)		log2(DGCR8)		log2(RAN)		log2(XPO5)		log2(NUP153)		log2(XPO1)		log2(DICER1)		log2(AGO2)	
	Ctrl	Sz	Ctrl	Sz	Crtl	Sz	Ctrl	Sz	Ctrl	Sz	Ctrl	Sz	Ctrl	Sz	Ctrl	Sz
**Valid**	23	28	23	28	23	28	23	28	23	28	23	28	23	28	23	28
**Missing**	0	0	0	0	0	0	0	0	0	0	0	0	0	0	0	0
**Mean**	8.667	8.575	6.671	6.451	10.456	10.483	5.658	5.450	8.887	8.936	7.589	7.562	9.513	9.523	8.612	8.613
**Std. Deviation**	0.120	0.188	0.478	0.590	0.391	0.354	0.672	0.789	0.191	0.186	0.324	0.363	0.336	0.284	0.171	0.194
**Minimum**	8.467	7.971	5.879	4.661	9.431	9.532	3.333	3.380	8.443	8.408	6.936	6.770	8.653	9.053	8.278	8.372
**Maximum**	8.877	8.893	7.591	7.412	10.887	11.053	6.589	6.534	9.255	9.417	8.130	8.210	10.472	10.217	8.968	9.296

Parietal cortex

	log2(DROSHA)		log2(DGCR8)		log2(RAN)		log2(XPO5)		log2(NUP153)		log2(XPO1)		log2(DICER1)		log2(AGO2)	
	Ctrl	Sz	Ctrl	Sz	Crtl	Sz	Ctrl	Sz	Ctrl	Sz	Ctrl	Sz	Ctrl	Sz	Ctrl	Sz
**Valid**	50	51	50	51	50	51	50	51	50	51	50	51	50	51	50	51
**Missing**	0	0	0	0	0	0	0	0	0	0	0	0	0	0	0	0
**Mean**	3.095	3.100	3.004	2.999	3.363	3.366	2.985	2.989	3.164	3.165	3.310	3.312	3.296	3.299	3.059	3.064
**Std. Deviation**	0.020	0.025	0.028	0.019	0.044	0.026	0.021	0.023	0.022	0.018	0.038	0.028	0.051	0.037	0.040	0.028
**Minimum**	3.037	3.028	2.970	2.955	3.183	3.304	2.921	2.939	3.065	3.117	3.175	3.244	3.230	3.233	2.989	2.984
**Maximum**	3.129	3.140	3.151	3.045	3.427	3.410	3.033	3.043	3.211	3.217	3.457	3.368	3.472	3.397	3.201	3.111

Superior Temporal cortex (BA22)

	log2(DROSHA)		log2(DGCR8)		log2(RAN)		log2(XPO5)		log2(NUP153)		log2(XPO1)		log2(DICER1)		log2(AGO2)	
	Ctrl	Sz	Ctrl	Sz	Crtl	Sz	Ctrl	Sz	Ctrl	Sz	Ctrl	Sz	Ctrl	Sz	Ctrl	Sz
**Valid**	19	23	19	23	19	23	19	23	19	23	19	23	19	23	19	23
**Missing**	0	0	0	0	0	0	0	0	0	0	0	0	0	0	0	0
**Mean**	7.640	7.680	5.441	5.614	9.045	9.080	6.004	5.980	7.484	7.577	6.351	6.504	8.680	8.715	7.675	7.766
**Std. Deviation**	0.199	0.172	0.381	0.254	0.505	0.395	0.225	0.213	0.179	0.213	0.338	0.350	0.383	0.294	0.181	0.188
**Minimum**	7.143	7.322	4.619	4.860	8.108	8.276	5.628	5.375	7.096	7.138	5.873	5.789	8.185	8.178	7.404	7.447
**Maximum**	7.846	8.099	6.137	5.986	9.692	9.676	6.409	6.418	7.706	7.941	7.172	7.239	9.387	9.400	8.076	8.325

Associative striatum

	log2(DROSHA)		log2(DGCR8)		log2(RAN)		log2(XPO5)		log2(NUP153)		log2(XPO1)		log2(DICER1)		log2(AGO2)	
	Ctrl	Sz	Ctrl	Sz	Crtl	Sz	Ctrl	Sz	Ctrl	Sz	Ctrl	Sz	Ctrl	Sz	Ctrl	Sz
**Valid**	18	18	18	18	18	18	18	18	18	18	18	18	18	18	18	18
**Missing**	0	0	0	0	0	0	0	0	0	0	0	0	0	0	0	0
**Mean**	10.108	9.987	7.767	7.884	11.230	10.944	7.645	7.586	9.695	9.594	8.994	9.512	10.701	11.025	6.890	7.173
**Std. Deviation**	0.135	0.299	0.171	0.228	0.271	0.471	0.159	0.228	0.093	0.197	0.292	0.454	0.573	0.434	0.313	0.355
**Minimum**	9.791	9.409	7.425	7.343	10.534	10.085	7.175	7.169	9.558	9.212	8.201	8.758	8.903	10.413	6.315	6.460
**Maximum**	10.314	10.525	8.089	8.219	11.694	11.767	7.901	8.053	9.899	10.052	9.390	10.283	11.679	11.717	7.450	7.893

Hippocampus

	log2(DROSHA)		log2(DGCR8)		log2(RAN)		log2(XPO5)		log2(NUP153)		log2(XPO1)		log2(DICER1)		log2(AGO2)	
	Ctrl	Sz	Ctrl	Sz	Crtl	Sz	Ctrl	Sz	Ctrl	Sz	Ctrl	Sz	Ctrl	Sz	Ctrl	Sz
**Valid**	18	15	18	15	18	15	18	15	18	15	18	15	18	15	18	15
**Missing**	0	0	0	0	0	0	0	0	0	0	0	0	0	0	0	0
**Mean**	8.225	8.093	6.110	6.301	10.629	10.190	5.209	5.001	7.898	7.903	6.598	6.941	9.215	9.593	8.153	8.389
**Std. Deviation**	0.199	0.154	0.246	0.255	0.320	0.423	0.236	0.235	0.133	0.134	0.216	0.460	0.292	0.354	0.200	0.173
**Minimum**	7.543	7.782	5.560	5.762	9.749	9.349	4.686	4.648	7.715	7.688	6.143	6.414	8.752	9.085	7.748	8.151
**Maximum**	8.441	8.315	6.574	6.612	11.000	10.863	5.539	5.410	8.128	8.215	6.928	8.091	9.828	10.204	8.511	8.617

DLPFC dorsolateral prefrontal cortex, *PFC* prefrontal cortex, *PBMCs* peripheral blood mononuclear cells, *BA* Brodmann area

Supplementary Table 3 (continued)

Cerebellum

	log2(DROSHA)		log2(DGCR8)		log2(RAN)		log2(XPO5)		log2(NUP153)		log2(XPO1)		log2(DICER1)		log2(AGO2)	
	**Ctrl**	**Sz**	**Ctrl**	**Sz**	**Ctrl**	**Sz**	**Ctrl**	**Sz**	**Ctrl**	Sz	Ctrl	Sz	Ctrl	Sz	Ctrl	Sz
**Valid**	50	44	50	44	50	44	50	44	50	44	50	44	50	44	50	44
**Missing**	0	0	0	0	0	0	0	0	0	0	0	0	0	0	0	0
**Mean**	3.227	3.234	3.220	3.226	3.366	3.364	3.130	3.137	3.326	3.339	3.453	3.458	3.392	3.400	3.154	3.166
**Std. Deviation**	0.016	0.013	0.018	0.020	0.014	0.015	0.020	0.019	0.017	0.015	0.013	0.021	0.011	0.010	0.022	0.019
**Minimum**	3.191	3.194	3.157	3.167	3.333	3.335	3.079	3.070	3.242	3.306	3.419	3.405	3.370	3.373	3.110	3.122
**Maximum**	3.260	3.257	3.253	3.260	3.397	3.406	3.170	3.173	3.350	3.382	3.491	3.512	3.415	3.419	3.210	3.202

Blood compartment (PBMCs)

	log2(DROSHA)		log2(DGCR8)		log2(RAN)		log2(XPO5)		log2(NUP153)		log2(XPO1)		log2(DICER1)		log2(AGO2)	
	**Ctrl**	**Sz**	**Ctrl**	**Sz**	**Ctrl**	**Sz**	**Ctrl**	**Sz**	**Ctrl**	Sz	Ctrl	Sz	Ctrl	Sz	Ctrl	Sz
**Valid**	29	43	29	43	29	43	29	43	29	43	29	43	29	43	29	43
**Missing**	0	0	0	0	0	0	0	0	0	0	0	0	0	0	0	0
**Mean**	3.158	3.144	2.898	2.876	3.299	3.297	2.941	2.916	3.380	3.359	2.710	2.716	2.213	2.259	2.615	2.585
**Std. Deviation**	0.020	0.021	0.040	0.035	0.015	0.019	0.032	0.032	0.022	0.032	0.096	0.061	0.077	0.086	0.043	0.057
**Minimum**	3.114	3.076	2.825	2.807	3.266	3.251	2.884	2.824	3.331	3.286	2.385	2.615	2.061	2.077	2.542	2.472
**Maximum**	3.198	3.193	2.999	2.978	3.339	3.354	2.986	2.973	3.423	3.425	2.876	2.845	2.352	2.488	2.724	2.791

Olfactory epithelium

	log2(DROSHA)		log2(DGCR8)		log2(RAN)		log2(XPO5)		log2(NUP153)		log2(XPO1)		log2(DICER1)		log2(AGO2)	
	**Ctrl**	**Sz**	**Ctrl**	**Sz**	**Ctrl**	**Sz**	**Ctrl**	**Sz**	**Ctrl**	Sz	Ctrl	Sz	Ctrl	Sz	Ctrl	Sz
**Valid**	19	19	19	19	19	19	19	19	19	19	19	19	19	19	19	19
**Missing**	0	0	0	0	0	0	0	0	0	0	0	0	0	0	0	0
**Mean**	3.387	3.377	2.944	2.864	3.526	3.563	2.858	2.865	3.275	3.284	2.954	2.718	3.620	3.606	3.565	3.542
**Std. Deviation**	0.035	0.021	0.307	0.322	0.047	0.029	0.082	0.053	0.045	0.056	0.180	0.219	0.021	0.014	0.023	0.028
**Minimum**	3.316	3.343	2.216	1.823	3.441	3.521	2.735	2.766	3.203	3.160	2.536	2.282	3.577	3.578	3.523	3.492
**Maximum**	3.449	3.423	3.272	3.208	3.606	3.632	3.002	2.998	3.342	3.382	3.244	3.031	3.646	3.633	3.609	3.611

Skin fibroblats

	log2(DROSHA)		log2(DGCR8)		log2(RAN)		log2(XPO5)		log2(NUP153)		log2(XPO1)		log2(DICER1)		log2(AGO2)	
	**Ctrl**	**Sz**	**Ctrl**	**Sz**	**Ctrl**	**Sz**	**Ctrl**	**Sz**	**Ctrl**	Sz	Ctrl	Sz	Ctrl	Sz	Ctrl	Sz
**Valid**	20	20	20	20	20	20	20	20	20	20	20	20	20	20	20	20
**Missing**	0	0	0	0	0	0	0	0	0	0	0	0	0	0	0	0
**Mean**	2.795	2.806	2.543	2.545	3.057	3.037	2.797	2.827	3.007	3.016	2.897	2.915	2.923	2.955	2.951	2.959
**Std. Deviation**	0.056	0.036	0.064	0.042	0.077	0.053	0.093	0.079	0.057	0.054	0.060	0.053	0.112	0.077	0.078	0.041
**Minimum**	2.695	2.721	2.389	2.462	2.893	2.956	2.566	2.656	2.905	2.893	2.792	2.793	2.619	2.769	2.810	2.874
**Maximum**	2.948	2.873	2.658	2.621	3.216	3.141	2.931	2.920	3.134	3.102	2.973	3.006	3.047	3.071	3.128	3.018

DLPFC (BA46) validation dataset

	log2(XPO1)		log2(DICER1)	
	**Ctrl**	**Sz**	**Ctrl**	**Sz**
**Valid**	29	30	29	30
**Missing**	0	0	0	0
**Mean**	6.603	6.935	9.905	10.188
**Std. Deviation**	0.462	0.730	0.277	0.460
**Minimum**	5.414	5.678	9.175	9.551
**Maximum**	7.245	9.375	10.527	11.151

Blood compartment (whole blood) validation dataset

	log2(DGCR8)		log2(XPO5)		log2(NUP153)		log2(DICER1)		log2(AGO2)	
	**Ctrl**	**Sz**	**Ctrl**	**Sz**	**Ctrl**	**Sz**	**Ctrl**	**Sz**	**Ctrl**	**Sz**
**Valid**	96	106	96	106	96	106	96	106	96	106
**Missing**	0	0	0	0	0	0	0	0	0	0
**Mean**	8.320	8.195	8.139	8.032	8.393	8.375	7.732	7.785	9.620	9.520
**Std. Deviation**	0.301	0.233	0.130	0.132	0.280	0.258	0.179	0.174	0.275	0.339
**Minimum**	7.754	7.561	7.841	7.695	7.669	7.627	7.398	7.354	8.863	8.767
**Maximum**	8.932	8.715	8.433	8.565	9.256	9.078	8.176	8.218	10.175	10.190

DLPFC dorsolateral prefrontal cortex, *PFC* prefrontal cortex, *PBMCs* peripheral blood mononuclear cells, *BA* Brodmann area

Supplementary Table 4 Genes with altered expression in the postmortem brain samples of individuals with schizophrenia relative to healthy controls

Gene	Primary datasets														Validation dataset	
	DLPFC (BA 46)		Anterior PFC (BA 10)		Parietal cortex		Superior temporal cortex		Associative striatum		Hippocampus		Cerebellum		DLPFC (BA 46)	
	Fold change	*adjusted p*-value[1]	Fold change	*adjusted p*-value[1]	Fold change	*adjusted p*-value[1]	Fold change	*adjusted p*-value[1]	Fold change	*adjusted p*-value[1]	Fold change	*adjusted p*-value[1]	Fold change	*adjusted p*-value[1]	Fold change	*adjusted p*-value[1]
DROSHA		NS		NS		NS		NS		NS	**0.91**	**0.010**	**1.03**	**0.048**		
DGCR8		NS		NS		NS		NS		NS	**1.14**	**0.042**		NS		
RAN		NS		NS		NS		NS		NS	**0.74**	**0.004**		NS		
XPO5		NS		NS		NS		NS		NS	**0.87**	**0.023**		NS		
NUP153		NS		NS		NS		NS		NS		NS	**1.06**	**0.002**		
XPO1	**1.32**	**0.008**		NS		NS		NS	**1.43**	**0.002**	**1.27**	**0.023**		NS		NS
DICER1	**1.13**	**0.008**		NS		NS		NS		NS	**1.3**	**0.005**	**1.04**	**0.003**	**1.22**	**0.010**
AGO2		NS		NS		NS		NS		NS	**1.18**	**0.004**	**1.05**	**0.021**		

DLPFC dorsolateral prefrontal cortex, *PFC* prefrontal cortex, *BA* Brodmann area

[1] Adjusted p-values set at 0.05

Supplementary Table 5 Genes with altered expression in the peripheral tissue samples of individuals with schizophrenia relative to healthy controls

Gene	**Primary datasets**						**Validation dataset**	
	Blood (PBMCs)		Olfactory epithelium		Skin fibroblasts		Blood (whole blood)	
	Fold change	*adjusted* *p*-value[1]	Fold change	*adjusted* *p*-value[1]	Fold change	*adjusted* *p*-value[1]	Fold change	*adjusted* *p*-value[1]
DROSHA	**0.94**	**0.013**		NS		NS		
DGCR8	**0.92**	**0.026**		NS		NS	**0.92**	**0.015**
RAN		NS	**1.23**	**0.019**		NS		
XPO5	**0.92**	**0.012**		NS		NS	**0.93**	**<0.001**
NUP153	**0.9**	**0.012**		NS		NS		
XPO1		NS	**0.45**	**0.007**		NS		
DICER1	**1.11**	**0.033**		NS		NS	**1.04**	**0.045**
AGO2	**0.92**	**0.016**	**0.87**	**0.019**		NS	**0.93**	**0.038**

PBMCs Peripheral blood mononuclear cells

[1] Adjusted p-values set at 0.05
